# Prevalence and antibiotic susceptibility of *Mannheimia haemolytica* and *Pasteurella multocida* isolated from ovine respiratory infection: A study from Karnataka, Southern India

**DOI:** 10.14202/vetworld.2020.1947-1954

**Published:** 2020-09-23

**Authors:** Swati Sahay, Krithiga Natesan, Awadhesh Prajapati, Triveni Kalleshmurthy, Bibek Ranjan Shome, Habibur Rahman, Rajeswari Shome

**Affiliations:** 1 Indian Council of Agricultural Research-National Institute of Veterinary Epidemiology and Disease Informatics, Bengaluru, Karnataka, India; 2 Department of Microbiology, Centre for Research in Pure and Applied Sciences, Jain University, Bengaluru, Karnataka, India; 3 International Livestock Research Institute, CG Centre, NASC Complex, DPS Marg, Pusa, New Delhi, India

**Keywords:** antimicrobial susceptibility, isolation, *Mannheima haemolytica*, multiplex PCR, *Pasteurella multocida*

## Abstract

**Background and Aim:**

Respiratory infection due to *Mannheimia haemolytica* and *Pasteurella multocida* are responsible for huge economic losses in livestock sector globally and it is poorly understood in ovine population. The study aimed to investigate and characterize *M. haemolytica* and *P. multocida* from infected and healthy sheep to rule out the involvement of these bacteria in the disease.

**Materials and Methods:**

A total of 374 healthy and infected sheep samples were processed for isolation, direct detection by multiplex PCR (mPCR), and antibiotic susceptibility testing by phenotypic and genotypic methods.

**Results:**

Overall, 55 Pasteurella isolates (27 [7.2%] *M. haemolytica* and 28 [7.4%] *P. multocida*) were recovered and identified by bacteriological tests and species-specific PCR assays. Significant correlation between the detection of *M. haemolytica* (66.6%) with disease condition and *P. multocida* (19.1%) exclusively from infected sheep was recorded by mPCR. *In vitro* antibiotic susceptibility testing of 55 isolates revealed higher multidrug resistance in *M. haemolytica* (25.9%) than *P. multocida* (7.1%) isolates. Descending resistance towards penicillin (63.6%), oxytetracycline (23.6%), streptomycin (14.5%), and gentamicin (12.7%) and absolute sensitivity towards chloramphenicol were observed in both the pathogens. The antibiotic resistance genes such as *strA* (32.7%) and *sul2* (32.7%) associated with streptomycin and sulfonamide resistance, respectively, were detected in the isolates.

**Conclusion:**

The study revealed the significant involvement of *M. haemolytica* together with *P. multocida* in ovine respiratory infection and is probably responsible for frequent disease outbreaks even after vaccination against hemorrhagic septicemia in sheep population of Karnataka, southern province of India.

## Introduction

Respiratory disease in ruminant population accounts for substantial economic losses to the livestock sector globally [1]. *Pasteurella multocida* and *Mannheimia haemolytica* are the main etiological agents of the disease known to cause 30% of deaths in feedlot cattle and acute outbreaks in sheep population resulting in huge mortality all across the world [2]. The clinical manifestations of the disease in small ruminants include dyspnea, pyrexia, dullness, reduced appetite, anorexia, rapid shallow respiration, profuse mucopurulent nasal, and ocular discharge and death within 12-24 h during the outbreaks [3]. *P. multocida* and *M. haemolytica* are Gram-negative, bipolar coccobacillus belonging to the family *Pasteurellaceae* of gamma proteobacteria [4,5]. Based on the capsular antigen, *M. haemolytica* has been classified into 12 serotypes (A1, A2, A5-A9, A12- A14, A16, and A17) [5] and based on capsular and somatic antigens, *P. multocida* is grouped into five serogroups (A, B, D, E, and F) and 16 serotypes [6].

The stress due to unfavorable environmental conditions, animal transportation and bacterial and viral infections are the predisposing factors for respiratory disease in ruminants [7]. The involvement of *P. multocida* and *M. haemolytica* in bronchopneumonia and presence of these bacteria as predictors of respiratory disease in ruminants are reported in various studies [8,9]. In ruminants, severity of respiratory infection leading to bronchopneumonia due to treatment failure as result of antimicrobial resistance has also been reported [10]. Various studies have noted the emergence of multidrug resistance to beta-lactams, tetracycline, streptomycin, sulfonamides, macrolides, and sulfamethazine in *M. haemolytica* and *P. multocida* [11,12].

Of the 512.05 million livestock population in India, the sheep and goats make up to 65.06 and 135.17 million, respectively [13]. Sheep and goats are extensively distributed all across the agro-ecological terrain of India contributing to the improvement of the socio-economic status of rural population [14]. Respiratory infection in sheep, the actual etiology and epidemiology are scarcely documented in the country. Frequent respiratory infection outbreaks in sheep even after vaccination against hemorrhagic septicemia (HS) in different parts of Karnataka, Southern India prompted us to investigate the etiology of the disease.

The study aimed to rule out the involvement of *P. multocida* and *M. haemolytica* in respiratory disease of sheep and to assess the phenotypic and genotypic antibiotic resistance for implementing appropriate therapeutic measures to control the disease.

## Materials and Methods

### Ethical approval

The study approved by Institutional Animal Ethics Committee, Indian Council of Agricultural Research-National Institute of Veterinary Epidemiology and Disease Informatics (ICAR- NIVEDI), Bengaluru, India and the authors have taken permission from the farm owners to publish the data. All applicable national, and institutional guidelines for the animal’s care were followed during the sample collection.

### Sample collection and processing

A total of 374 (nasal-242 and lung-132) samples were collected from five different districts of Karnataka state from April 2015 to December 2016 (Table-1). Among the nasal samples, 59 were collected from apparently healthy sheep from five different flocks with no history of respiratory infection before one month of sample collection. Similarly, 183 nasal samples were collected from sheep exhibiting symptoms of respiratory infection (nasal discharge, lacrimal discharge, fever, weakness, and diarrhea) from 11 different flocks. The deep nasal swabs samples were collected into 2 ml brain heart infusion (BHI) broth. Lung tissue samples (n=132 [healthy-94 and lung with lesions-38]) were collected while slaughtering sheep at Bengaluru Municipal Abattoir, Bengaluru, India, in a tissue collection containers and transported to the laboratory.

**Table-1:** Details of nasal and lung samples collected from different locations of Karnataka.

No.	Place	Latitude	Longitude	Name of district	Healthy samples	Infected samples	Total samples
1	Chikkajala	13.1715° N	77.6356° E	Bangalore Urban	13	35	48
2	Channahalli	13.1790° N	77.6165° E	Bangalore Rural	6	47	53
3	Seethakempanahalli	13.1729° N	77.5195° E	Bangalore	0	10	10
4	Chintamani	13.3862° N	78.0603° E	Kolar	9	0	9
5	Kolar	12.9984° N	78.0737° E	Kolar	0	42	42
6	Kalavara	13.5970° N	74.7483° E	Chikkaballapur	7	15	22
7	Gauribidanur	13.6112° N	77.5170° E	Chikkaballapur	24	34	58
8.	Municipal Abattoir Shivajinagar, Bangalore	12.9857° N	77.6057° E	Bangalore	94	38	132 (lung samples)
	Total number of samples				153	221	

### Bacterial isolation and identification

For isolation of *M. haemolytica* and *P. multocida*, nasal samples enriched for 18 h in BHI broth were inoculated onto Tryptic Soya Agar (TSA) supplemented with 5% sheep blood (blood agar) and 15 mg/mL of bacitracin and incubated at 37°C for 24 h [15]. For lung samples, the tissue near to the bronchus was directly inoculated onto blood agar with 15 mg/mL of bacitracin and incubated as mentioned above. The colonies obtained on blood agar were purified once again on blood agar and later on BHI agar. The colonies showing the morphology of *M. haemolytica* (β-hemolytic, white-grayish, medium-sized round, and non-mucoid) and *P. multocida* (non-hemolytic, greyish, medium-sized round, and non-mucoid) were processed separately for the identification by bacteriological tests (Gram’s staining, catalase, oxidase, indole, lactose fermentation, and growth on MacConkey lactose agar [MLA]) as per standard protocols [15].

### Species confirmation by PCR

The genomic DNA was extracted from pure cultures by DNeasy kit as per the manufacturer’s protocol (Qiagen, Hilden, Germany). The quality and quantity of the extracted DNA was ascertained by NanoDrop2000 (Thermo Scientific, Waltham, USA) and on 0.8% agarose gel electrophoresis. Isolates were confirmed by species-specific multiplex PCR (mPCR) assay targeting *lktD*, HP; NZ_AASA01000080 and *16S rDNA* [16] for *M. haemolytica* and species-specific PCR using KMT1SP6-KMT1T7 primers for *P. multocida* [17] (Table-2).

**Table-2:** List of the primers used for species identification, capsular, and antibiotics resistance gene typing of *M. haemolytica* and *P. multocida* isolates.

No.	Primer sets	Sequence (5’→3’)	Gene target	Fragment size (bp)	Reference
***M. haemolytica* identification**					

1	Sod A	(F)AGCAGCGACTACTCGTGTTGGTTCG (R)AAGACTAAAATCGGATAGCCTGAACGCTG	*sodA*	143	[18]
2	Lkt	(F)GCAGGAGGTGATTATTAAAGTGG (R)CAGCAGTTATTGTCATACCTGAAC	*lktD*	206	[16]
3	Lkt2	(F)CTCTCTTTAGAAAAGCTGGAAAC (R)TTTTGCCAAGTGGTGTATTGC	*lktD*	179	
4	HP	(F)CGAGCAAGCACAATTACATTATGG (R)CACCGTCAAATTCCTGTGGATAAC	Unknown/NZ_AASA01000080	90	
5	16S	(F)GCTAACTCCGTGCCAGCAG (R)CGTGGACTACCAGGGTATCTAAC	*16S rDNA*	304

***P. multocida* identification**					

6	KMT1T7 KMT1SP6	(F)ATC CGC TAT TTA CCC AGT GG (R)GCT GTAAAC GAACTC GCC	KMT1	460	[17]

**Antibiotics resistance gene typing**					

**No.**	**Antibiotics / gene**	**Sequence (5’→3’)**	**Annealing temperature (°C)**	**Fragment size (bp)**	**Reference**
14	Tetracyclin/*tetH*	(F)ATACTGCTGATCACCGT (R)TCCCAATAAGCGACGCT	60	1076	[20]
15	Oxytetracycline/*ICE tetR*	(F)CGGCTTGGGTTAATAATGGCG (R)ATAACGCGAAAAGCTTCCGC	58	425	
16	Penicillin/*bla*_OXA-2_	(F)GCAGACGAACGCCAAGCGGA (R)CCCGCACGATTGCCTCCCTC	64	625	
17	Ampicillin/*bla*_ROB-1_	(F)AATAACCCTTGCCCCAATTC (R)TCGCTTATCAGGTGTGCTTG	60	685	
18	Sulfonamide/*sul2*	(F)CCAATACCGCCAGCCCGTCG (R)TGCCTTGTCGCGTGGTGTGG	64	489	
19	Gentamicin/*aadB*	(F)TTACGCAGCAGGGCAGTCGC (R)GCGGCACGCAAGACCTCAAC	66	551	
21	Streptomycin/*strA/strB*	(F)AAGGCAAGGCGTTCGCGGTC (R)CCGGCGGCTGATCTGTCTGG	64	506/586	
		(F)TCGCACCTGCTTGATCGCGG (R)GCTCGAATATGCCGGGGAGCG			
20	Chloramphenicol/*catAIII*	(F)ACCATGTGGTTTTAGCTTAACA (R)GCAATAACAGTCTATCCCCTTC	64	470	[21]

M. haemolytica=Mannheimia haemolytica, P. multocida=Pasteurella multocida

### Direct detection of *P. multocida* and *M. haemolytica* by mPCR

The mPCR was standardized for the rapid detection of *P. multocida* and *M. haemolytica* species using set of primers for *P. multocida* (KMT1SP6-KMT1T7 [460bp]) [17] and *M. haemolytica* (*sodA* gene primer for 143bp) [18] (Table-2). The DNA was extracted from samples enriched in BHI broth for 18 h by DNeasy kit as per the manufacturer’s protocol (Qiagen, Hilden, Germany). Extracted DNA (100ng/μl) served as template in 25 μl PCR mixture consisting of 1× hotstart Taq plus master mix (Thermo scientific) and 0.3 μM and 0.5 μM of *M. haemolytica* and *P. multocida* primers, respectively. PCR amplification was performed using mastercycler (Eppendorf Ltd., Mississauga, Canada) with the following thermal conditions: Initial denaturing temperature of 95°C for 5 min followed by 30 cycles of 94°C for 30 s, annealing temperature of 56°C for 1 min, extension temperature of 72°C for 1 min, and final extension at 72°C for 8 min. The PCR products were analyzed by electrophoresis on 1.5% agarose gel with 10 μg/ml^-1^ ethidium bromide and visualized in gel documentation system. *M. haemolytica* serotype A2 (ATCC No. 29698) and serotype A7, (ATCC No. 33396) and *P. multocida* type A (ATCC No. 12945) and type B serogroups (ATCC No. 43137) were used as positive controls.

### *In vitro* antimicrobial susceptibility test (ABST)

*In vitro* ABST was performed for 28 *P. multocida* and 27 *M. haemolytica* isolates using Kirby–Bauer disk diffusion method [19] on Muller-Hinton agar as per Clinical and Laboratory Standards Institute (CLSI) with *Escherichia coli* ATCC 25922 as a quality control organism. The antibiotics commonly used in the treatment of the respiratory ailments were chosen, namely, amoxicillin/clavulanic acid (30 mcg), co-trimoxazole (25 mcg), ampicillin (10 mcg), penicillin (10 U), enrofloxacin (5 mcg), chloramphenicol (30 mcg), gentamicin (10 mcg), streptomycin (10 mcg), oxytetracycline (30 mcg), and tetracycline (30 mcg). The zone of inhibition was interpreted as per the performance standards for ABST specified in 16 informational supplement of CLSI (CLSI, 2006).

### Antibiotic resistance marker genotyping

All the isolates irrespective of the resistance pattern in ABST were subjected to antibiotic resistance marker typing for penicillin (*bla*_OXA-2_), ampicillin (*bla*_ROB-1_), sulfonamide (*sul2*), gentamicin (*aadB*), chloramphenicol (*CatAII*), tetracycline (*tet H*), and streptomycin (*strA, strB*) by simplex PCR assays [20,21] (Table-2).

### Statistical analysis

Statistical analysis was performed with SPSS 16.0 (SPSS Inc., Chicago) and p<0.05 was considered statistically significant.

## Results

### Isolation and identification of *M. haemolytica* and *P. multocida*

**Figure-1:**
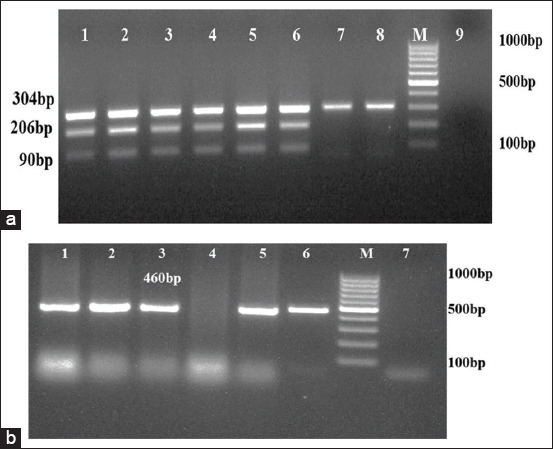
(a) Multiplex PCR amplification for *Mannheimia haemolytica*. Lane 3-8: DNA extracted from pure cultures; Lane 1-2: Positive controls; Lane 9: No template control; Lane M: 100bp molecular marker; (b) PCR amplification for *Pasteurella* *multocida.*Lane1-5: DNA extracted from field isolates; Lane 6: Positive control; Lane 7: No template control; Lane M: 100bp molecular marker.

Among 374 samples processed, 64 (17.1%) and 35 (9.4%) isolates obtained were identified as *M. haemolytica* and *P. multocida*, respectively, by bacteriological tests (Table-3). All of these isolates were Gram-negative, showed bipolar coccobacilli morphology microscopically and positive to oxidase and catalase tests. *M. haemolytica* isolates showed β-hemolysis on blood agar, variable lactose fermentation reaction on MLA and negative for indole production. Whereas, *P. multocida* isolates were non-hemolytic, indole positive and failed to grow on MLA. Among these identified isolates, 27 were confirmed as *M. haemolytica* by amplification of 304, 206, and 90 bps in mPCR (Figure-1a) and 28 isolates as *P. multocida* by amplification of 460bp (Figure-1b). From 132 lung tissue samples processed, 11 and 14 isolates of *M. haemolytica* and *P. multocida*, respectively, were recovered from infected lungs showing a significant correlation between isolation and disease status (p<0.0001). Similarly, 13 *P. multocida* isolates were recovered solely from the nasal samples of sheep suffering from respiratory infection. Co-isolation of *M. haemolytica* and *P. multocida* was observed in 15.7% infected lung samples (Table-4).

**Table-3:** Isolation and mPCR detection of *M. haemolytica* and *P. multocida* from various types of sheep samples.

Type of samples	Number of samples	*M. haemolytica*				*P. multocida*			
	
		mPCR positives	χ^2^/p-value	Number of isolates	χ^2^/p-value	mPCR positives	χ^2^/p-value	Number of isolates	χ^2^/p-value
Nasal samples	242	143 (59)	0/1	14 (5.8)	1.80/ 0.179	35 (14.5)	0.91/ 0.340	13 (5.4)	3.75/ 0.052
Lung samples	132	78 (59)		13 (9.8)		25 (18.9)		15 (11.3)	
Total samples	374	221 (59)		27 (7.2)		60(16.0)		28 (7.5)	
Nasal samples									
Nasal samples from healthy animals	59	21 (35.6)	5.13/0.023*	2 (3.4)	0.74/ 0.389	0		0	
Nasal samples from respiratory infection	183	122 (66.7)		12 (6.6)		35 (19.1)		13(7.1)	
Total samples	242	143 (59)		14 (5.8)		35 (14.5)		13(5.4)	
Lung samples									
Healthy lungs	94	57 (60.6)	0.08/0.777	2 (2.1)	16.48/<0.0001*	22 (23.4)	3.07/0.079	1 (1.1)	24.55/<0.0001*
Infected lungs	38	21 (55.3)		11 (28.9)		3 (7.9)		14 (36.8)	
Total samples	132	78 (59)		13 (9.8)		25 (18.9)		15 (11.4)	

The values within the parentheses indicates percentage;

* Significance at p<0.05

**Table-4:** Concurrence of *M. haemolytica* and *P. multocida* by isolation and mPCR in nasal and lung sheep samples.

Samples	Total samples	mPCR co-positives	Co-isolations
Total nasal samples	242	29 (12)	0
Total lung samples	132	25 (18.9)	6 (4.5)
Cumulative total samples	374	54 (14.4)	6 (1.6)
Nasal samples			
Nasal samples from healthy sheep	59	0	0
Nasal samples from sheep with respiratory infection	183	29 (15.8)	0
Total samples	242	29 (12)	
Lung samples			
Healthy lung samples	94	22 (23.4)	0
Infected lung samples	38	3 (7.9)	6 (15.8)
Total samples	132	25 (18.9)	6 (4.5)

The values within the parentheses indicate percentage. *M. haemolytica=Mannheimia haemolytica, P. multocida=Pasteurella multocida,* mPCR=Multiplex PCR

### Direct detection of *M. haemolytica* and *P. multocida* by mPCR

**Figure-2:**
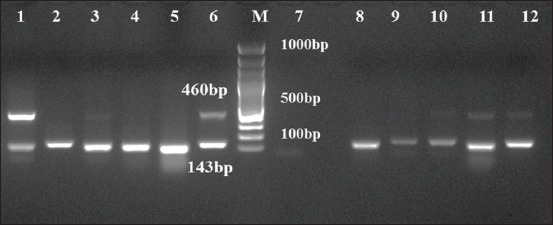
Multiplex PCR amplification for *Mannheimia haemolytica* and *Pasteurella multocida*. Lane1-5 and 8-12: DNA extracted from 18 h BHI broth enriched samples; Lane 6: Positive control; Lane 7: No template control; Lane M: 100bp molecular marker.

Out of 374 samples processed, 221 (59%) and 60 (16.04%) samples were positive for *M. haemolytica* and *P. multocida*, respectively, by mPCR. Of which 66.6% *M. haemolytica* and 19.1% *P. multocida* were from nasal samples collected from sheep suffering from respiratory infection. Among 94 and 38 healthy and infected lungs samples processed respectively, higher percentage of both *M. haemolytica* (60.6%) and *P. multocida* (23.4%) were detected in healthy lungs (Table-3 and Figure-2) and co-detection of both *M. haemolytica* and *P. multocida* was observed in 18.9% of lung samples (Table-4).

### *In vitro* antimicrobial susceptibility testing

**Figure-3:**
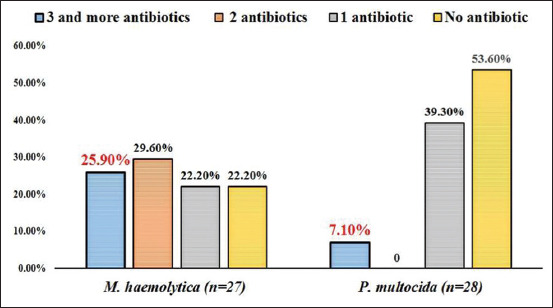
Multi drug resistance profile in *Mannheimia haemolytica* and *Pasteurella multocida* isolates.

Among ten different antibiotics tested, chloramphenicol (100%), ampicillin (98.9%), and amoxicillin/clavulanic acid (96.4%) were found most effective drugs against *M. haemolytica* and *P. multocida* (Table-5). *M. haemolytica* isolates showed 81.5%, 40.7%, and 22.2% resistance towards penicillin, oxytetracycline, and streptomycin, respectively, whereas, *P. multocida* isolates showed resistance only towards penicillin (46.4%) and 7.1% each for gentamicin, oxytetracycline, and streptomycin. Among 55 isolates tested, 22 (81.5%) and 13 (46.4%) isolates of *M. haemolytica* and *P. multocida*, respectively, were resistant to at least one antibiotic. Multiple drug resistance against three or more than three antibiotics was noticed in 7.1% (2/28) of *P. multocida* and 25.9% (7/27) of *M. haemolytica* isolates (Figure-3).

**Table-5:** *In vitro* antibiotic susceptibility of *P. multocida* and *M. haemolytica* isolates from sheep.

No.	Antibiotics (Conc./disc)	*P. multocida* (n=28)		*M. haemolytica* (n=27)		Total (n=55)	
		
		Resistant	Intermediate resistant	Resistant	Intermediate resistant	Resistant	Intermediate resistant
1	Amoxicillin/clavulanic acid (30 mcg)	1 (3.6)	0	1 (3.7)	0	2 (3.6)	0
2	Co-trimoxazole (25 mcg)	1 (3.6)	0	4 (14.8)	0	5 (9.0)	0
3	Ampicillin (10 mcg)	0	0	0	1 (3.7)	0	1 (1.8)
4	Penicillin (10 U)	13 (46.4)	0	22 (81.5)	0	35 (63.6)	0
5	Enrofloxacin (5 mcg)	0	2 (7.1)	0	13 (48.1)	0	15 (27.2)
6	Chloramphenicol (30 mcg)	0	0	0	0	0	0
7	Gentamicin (10 mcg)	2 (7.1)	1(3.5)	5 (18.5)	4 (14.8)	7 (12.7)	5 (9.0)
8	Streptomycin (10 mcg)	2 (7.1)	1(3.5)	6 (22.2)	11 (40.7)	8 (14.5)	12 (21.8)
9	Oxytetracycline (30 mcg)	2 (7.1)	0	11 (40.7)	0	13 (23.6)	0
10	Tetracycline (30 mcg)	0	0	3 (11.1)	0	3 (5.5)	0

The values within the parentheses indicate percentage. *M. haemolytica=Mannheimia haemolytica, P. multocida=Pasteurella multocida*

### Antibiotic resistance marker typing

Antibiotic resistance genes by PCR screening did not show any agreement with phenotypic *in vitro* antibiotic resistance profiles. However, the resistant genes for different antibiotics were higher in *M. haemolytica* compared to *P. multocida* isolates similar to phenotypic *in vitro* method. Out of 28 *P. multocida* isolates, 14 and 12 isolates showed the presence of *strA and sul2* genes. Among 27 *M. haemolytica* isolates, one, three, four, and six isolates showed the presence of *bla*_ROB-1_*, bla*_OXA-2_*, strA*, and *sul2* genes, respectively (Table-6).

**Table-6:** Detection of antibiotic resistance gene markers in *P. multocida* and *M. haemolytica* isolates.

*M. haemolytica* isolates		

Antibiotic resistance markers	No. of positive isolates in nasal samples (n=14)	No. of positive isolates in lung samples (n=13)
Penicillin (*bla*_OXA-2_)	3 (21.4)	0
Ampicillin *(bla*_ROB-1_*)*	1 (7.1)	0
Streptomycin (*strA, strB)*	1 (7.1) *(strA)*	3 (23)
Chloramphenicol (*catAII)*	0	0
Tetracycline (*tetH)*	0	0
Sulfamethoxazole (*sul2)*	2 (14.3)	4 (30.8)
Gentamicin (*aadB)*	0	0

***P. multocida* isolates**		

**Antibiotic resistance markers**	**No. of positive isolates in nasal samples (n=13)**	**No. of positive isolates in lung samples (n=15)**

Penicillin (*bla*_OXA-2_)	0	0
Ampicillin (*bla*_ROB-1_)	0	0
Streptomycin (*strA, strB*)	5 (38.5) *(strA)*	9 (60) *(strA)*
Chloramphenicol (*catAII*)	0	0
Tetracycline (*tetH*)	0	0
Sulfamethoxazole (*sul2*)	3 (23)	9 (60)
Gentamicin (*aadB*)	0	0

The values within the parentheses indicate percentage. *M. haemolytica=Mannheimia haemolytica, P. multocida=Pasteurella multocida*

## Discussion

Small ruminants are the continuous source of income for the rural populace in India. Increasing prevalence of respiratory infection in small ruminants even after HS and PPR vaccination was a serious concern. Recently respiratory infection outbreaks due to *M. haemolytica* and *P. multocida* have been reported in various states of India [22,23]. In the present study, we investigated the involvement of *M. haemolytica* and *P. multocida* with the respiratory infection of sheep.

Overall, *Pasteurella* species recovered from samples were 55 (14.7%), of which, 51% and 49% were identified as *P. multocida* and *M. haemolytica*, respectively, and majority of the isolates were from sheep suffering from respiratory infection. Similarly, Miller *et al*. [9] noted 80% biovariants of *M. haemolytica*, *P. multocida* and *P. trehalosi* species from the diseased sheep indicating the importance of these pathogens in the respiratory infections. In mPCR, *M. haemolytica* was detected in 59% of the samples and significant correlation was observed between *M. haemolytica* detection (66.7%) with the diseased condition (p=0.023) which substantiates its role in the disease. Whereas, 35.6% of *M. haemolytica* PCR positives were also detected in nasal samples collected from apparently healthy sheep. This may be because of the fact that *M. haemolytica* is generally present in the upper respiratory tract of ruminants which multiplies rapidly along with *P. multocida* upon exposure to the stress thereby resulting in respiratory illness [7]. Compared to *M. haemolytica*, the detection of *P. multocida* by mPCR was less (16.04%) but was detected solely from the infected sheep (14.4%) which acknowledges the primary role of *P. multocida* in respiratory infection. The pathogenic role of *P. multocida* in ovine pasteurellosis causing serious outbreaks was reported earlier [2]. Usually, in stressed and immunocompromised host, these secondary bacterial pathogens proliferate and increase in number in the upper respiratory tract and by gravitational drainage they reach to the ventral bronchi, bronchioles, and alveoli to cause bronchopneumonia [7]. Therefore we tried to detect these pathogens in lungs samples by mPCR. *M. haemolytica* was detected in both infected and healthy lung samples, whereas the higher number of *P. multocida* were recorded in healthy lung samples. The correlation of the disease status of the slaughtered animals to the detection of the pathogens was difficult as the animals were under transportation stress and their clinical symptoms were unknown.

As a result of ineffective immunoprophylactic measure for respiratory infection, antimicrobial treatment is considered important prophylactic agent for the control of the disease. Due to extensive use of antibiotics as supplements in animal feed both for prophylaxis and growth promotion, antimicrobial resistance among these pathogens was observed [11]. Among ten different antibiotics tested, chloramphenicol (100%), ampicillin (98.9%), and amoxicillin/clavulanic acid (96.4%) were found the most effective drugs against *M. haemolytica* and *P. multocida* isolates. In the present work, *M. haemolytica* and *P. multocida* isolates showed only 18.5% and 7.1% resistance to gentamicin, respectively. Whereas, the study from Ethiopia reported gentamycin as totally inactive against *M. haemolytica* and *P. multocida* isolates for the treatment of ovine pasteurellosis [24]. Lamm *et al*. [10] reported high resistance to tetracycline in *M. haemolytica* and *P. multocida* isolates from bronchopneumonic cattle, whereas, only 11.1% of ovine *M. haemolytica* isolates were resistant to tetracycline in the present study. Klima *et al*. [15] noted high resistance towards oxytetracycline, ampicillin, and amoxicillin/clavulanic acid among bovine *M. haemolytica* isolates. Similarly in our study, ovine *M. haemolytica* isolates showed 40.7% resistance to oxytetracycline with least resistance to ampicillin and amoxicillin/clavulanic acid. Absolute sensitivity towards chloramphenicol and absolute resistance towards sulfa drug in avian *P. multocida* stains was reported from India [25]. However, in the present study, absolute sensitivity towards both chloramphenicol and sulfa drugs was observed. Multiple drug resistance (three or more than three antibiotics) was detected higher in *M. haemolytica* than *P. multocida* isolates. Andrés-Lasheras *et al*. [26] and Sarangi *et al*. [27] have also noted multiple drug resistance in *M. haemolytica* isolates from BRD infected European cattle and Indian *P. multocida* isolates from small ruminants. So, periodical antibiotic susceptibility testing is essential to identify the drug/s of choice for the treatment in different host/s and to set guidelines for the prudent use of antibiotic/s in the disease endemic regions.

Along with the external factors such as geographical location, antibiotic pre-treatment, and dosages, antibiotic resistance among *Pasteurella* species also depends on accessibility of the isolates to the resistance genes in the gene pool. Genes such as *bla*_ROB-1,_ *tetH*, *tetO*, *tetB*, and *strA* associated with β-lactam, tetracycline, and streptomycin resistance, respectively, were detected in *P. multocida* pig isolates from Spain [28]. The *tetH*, *bla*_ROB-1_ genes were also identified in *M. haemolytica* isolates from cattle treated for BRD [15]. In the present study, *bla*_OXA-2_*, bla*_ROB-1_*, strA*, and *sul 2* genes in *M. haemolytica* isolates and only *strA* and *sul2* resistance genes in *P. multocida* isolates were observed. Plasmids [28], chromosome [11], and integrative conjugative elements [29,30] are the associated factors for the interspecies and inter-genic antibiotic resistance genes transmission in *Pasteurellaceae* family. Presence of different antibiotic resistance genes in *M. haemolytica* is as serious concern as these genes can be transferred to *P. multocida* and other respiratory pathogens by horizontal gene transfer which may cause severe infections.

## Conclusion

The study emphasized the significance of *M. haemolytica* together with *P. multocida* in causing respiratory infection in sheep. The study suggests to include *M. haemolytica* while diagnosing respiratory disease which has been ignored or overlooked. Higher drug resistance in *M. haemolytica* than *P. multocida* together with the presence of *strA* and *sul2* like antibiotic resistance genes was observed in the isolates. Further studies involving samples from multiple hosts from different geographical and anatomic locations are needed to understand the serotypic, pathogenic, and genotypic variants causing the disease outbreaks in the country.

## Authors’ Contribution

RS and SS were involved in planning, execution of the work, and writing of the manuscript. SS, KN, AP, and TK performed disease investigation, sample collection, bacterial isolation, and characterization. BRS and HR provided the conceptual and technical support to formulate the work. All authors read and approved the final manuscript.
